# Relationship of ulna styloid fracture to the distal radio-ulnar joint stability. A clinical, functional, and radiographic outcome study

**DOI:** 10.1371/journal.pone.0279210

**Published:** 2023-01-20

**Authors:** Vivek Ajit Singh, Tan Yong Jia, Rupini Devi Santharalinggam, Jayaletchumi Gunasagaran

**Affiliations:** Faculty of Medicine, Department of Orthopaedic Surgery (NOCERAL), University of Malaya, Kuala Lumpur, Malaysia; Saint George’s Hospital Medical School: St George’s University of London, IRAQ

## Abstract

**Background:**

Ulna styloid fracture occurs approximately about 55% of all distal end of radius fractures. However, the clinical and functional outcome of these fractures remains indefinite.

**Results:**

Only 56 patients with distal radius fractures had concomitant ulna styloid fractures. The mean age was 32 years (range: 18–69; SD: ± 12.7). The majority were men. The mean time from injury was 18.7 months (range: 6–84; SD: ± 13.3). The most common was Frykman 2, followed by 6, type 8, and type 4. All were closed fractures; 60.7% were base, and 39.3% were tip fractures. 50% were treated with casting, 48.3% plating, and 1.8% external fixation. The mean period of casting was 7.67 weeks (range: 4–16; SD ± 3.1). The ulna styloid was united in 35.7%. There is no significant difference in the range of movement between those with ulna styloid union and non-union. The Ballottement test and Piano key sign was statistically insignificant between both groups. All the displacements were dorsal except in 1 case. The mean displacement of ulna styloid is 1.88mm (SD±1.08, Range: 0.20–4.60mm). The mean VAS score at rest and work is not statistically significant. The mean grip strength and functional score (DASH) are similar in both groups.

**Conclusion:**

Ulna styloid fractures do not contribute to the DRUJ instability and the status of the union of the ulna styloid and the site of the ulna styloid fracture (tip or base) did not have a bearing on the range of movement and functional status of the affected wrist. Temporary DRUJ immobilization might allow TFCC recovery.

## Introduction

Ulnar styloid fractures are invariably associated with distal radius fractures and occur in about 55% of all distal radius fractures [1]. However, its contribution to the clinical and functional outcome of these fractures remains unclear [2]. The distal radioulnar joint (DRUJ) is a complex synovial-lined pivot joint formed by the sigmoid notch of the distal end radius, which receives the ulna head [3]. Anatomically, the DRUJ has a significant role in maintaining wrist stability, especially during forearm rotation. The bony architecture of the joint accounts for only 20% of its stability, while the remaining are contributed by both intrinsic and extrinsic soft tissue stabilizers. Ulna styloid provides an important ulnar-sided anchor for the triangular fibrocartilage complex (TFCC) components, particularly at the base of the ulna styloid. Its importance is further confirmed by anatomic and biomechanical studies [2]. Therefore, ulnar styloid fractures may alter the functional outcome of distal radius fractures, leading to instability, disability, and pain [4–10].

DRUJ instability can be easily missed clinically or on radiographs. Hence, a high index of suspicion and adequate understanding of the wrist anatomy is required. With the above-mentioned possible complications, it may seem that surgical stabilization of the fractured ulna styloid is the only wise option to address potential wrist disability [11]. However, there is a continuous debate on the effect of ulna styloid fracture on the stability of the wrist and its management.

This study evaluates the wrist’s clinical, functional, and radiographic outcomes in different ulna styloid fractures and the need for ulna styloid fixation during the primary distal radius fixation.

## Materials and methods

This is a retrospective study. All patients treated for distal end radius fractures with concomitant ulna styloid fractures at least six months after the injury and who visited the Orthopaedic Clinic at our center from Jun 1, 2018, to Jun 30, 2019, were recruited. The radius fracture management varied from casting, external fixation, and plating. All the ulna styloid fractures were not treated surgically. Patients with pathological fractures, history of the previous fracture, injury or surgery on the affected wrist, skeletally immature bone, concomitant ipsilateral upper limb fractures, bilateral concurrent wrist fractures, and non-union of the ipsilateral radius fracture were excluded from the study.

Medical review and ethics committee’s approval was obtained to conduct this study (MRECID.NO: 2017722–5424). Written informed consent was obtained from all participants.

All participants were interviewed concerning the fracture and the treatment they had received. The information was confirmed with their past medical records, and they were required to complete the DASH questionnaire. Subsequently, radiographs of their affected wrist in true anteroposterior (AP) and lateral views were taken. Based on the available radiographs, the fractures were classified based on the Frykman classification of distal radial fractures (2,4,6 and 8). Three investigators conducted the clinical assessments and radiographs measurements and the final outcome is based on the agreement of all three investigators.

### Clinical assessment

1. **DRUJ Ballottement Test (Holding technique):** It is a passive mobility test to assess the dorso-palmar laxity of the DRUJ with the forearm in the neutral position. The laxity was compared between both wrists. The outcome is considered positive when there is a displacement of the ulnar head or a lack of end-point resistance on the radius.
2. **Piano key sign:** This test is conducted in 2 positions, forearm supination and pronation. It detects either palmar or dorsal radio-ulnar ligament disruption by identifying abnormal translation of the ulnar head to the distal radius.
3. **Range of movement:** Ranges of motion of both wrists are measured using a standard goniometer. The wrist’s six-movement axes (Pronation and Supination, Ulnar and Radial deviation, Extension and Flexion) are measured. The measurements are recorded in degrees and registered as a percentage compared to the normal side.

### Radiograph assessment

**Fig 1:**
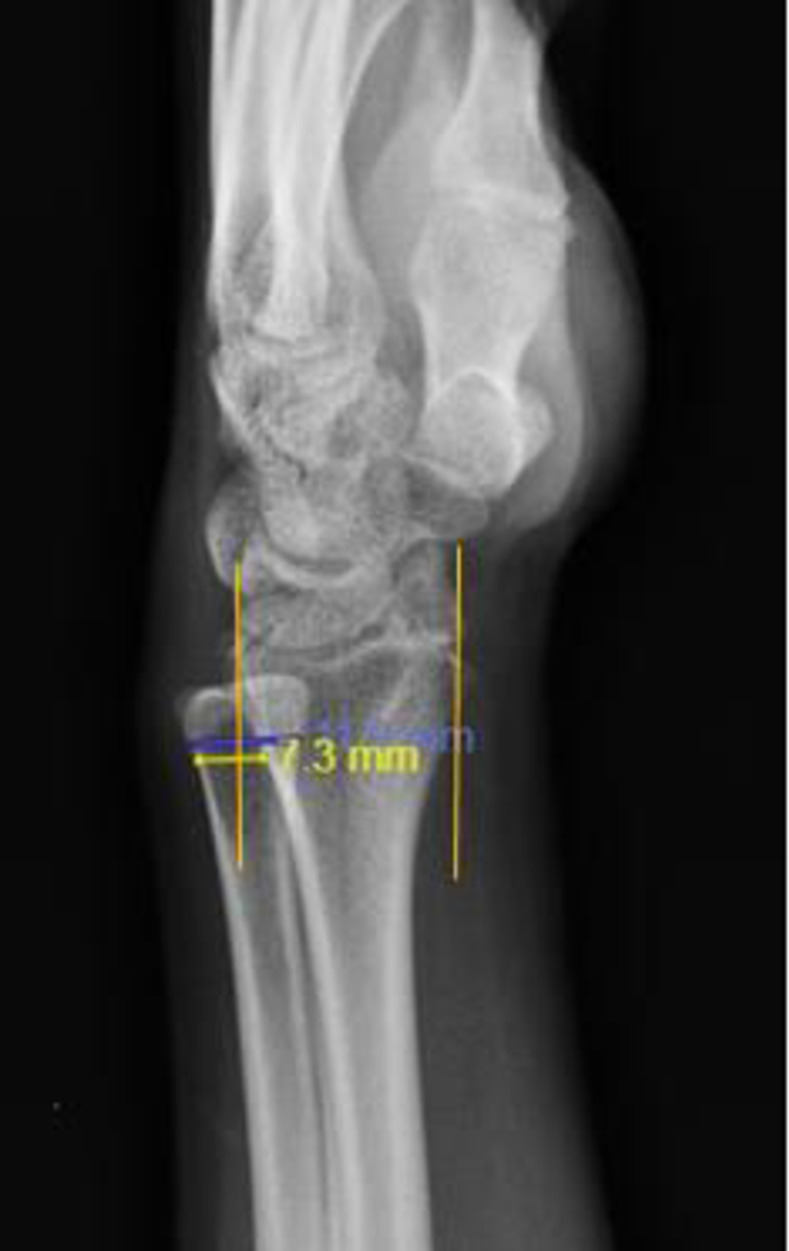
A line is drawn through both the volar and dorsal margins of the distal radius which is extended through the ulnar head. Instability is present when more than 25% of the head of the ulna lies volar or dorsal to these lines. Plain radiograph of Mr. M (Study subject) showing dorsal subluxation of the DRUJ using the Mino Method. 7.3mm/11.9mm x 100% = 61.34%.

1. **Union status:** union is defined as bridging callus across the fracture site at least 3 out of 4 cortices on AP and lateral views plain radiographs.
2. **Degree of displacement of the ulna styloid fracture fragment:** the distance between the fractured non-united ulna styloid with the original location is measured on the AP radiograph by the unit millimeter (mm).
3. **DRUJ subluxation/ dislocation:** Based on the Mino method, two lines were drawn through both the volar and dorsal margins of the distal radius in lateral view radiographs (Fig 1), extended through the ulnar head. Instability is present when more than 25% of the ulna head lies volar or dorsal to these lines. It is described by David E. Mino et al. to diagnose DRUJ subluxation radiographically [12].

### Functional assessment

1. **Ulnar-sided Wrist Pain:** This assessment indicates triangular fibrocartilage complex (TFCC) injury. The palpation area is at the depression between the flexor carpi ulnaris (FCU) tendon, ulnar styloid, and the triquetrum bone. The positive fovea sign carries 95% sensitivity and 87% specificity for foveal disruption/ ulnotriqeutral ligament injuries [13]. The pain is graded using the Visual Analog Scale (VAS) at rest and work (minimum 3kg weight).
2. **Grip Strength:** Grip strength is tested by using the hand-held Jamar dynamometer. Both hands were tested and compared; 3 attempts in each hand. The highest value from the three attempts is recorded in kilograms (Kg). For this test, the patient is seated in an upright position, and the device is being held with the testing limb in 90° forward flexion of the shoulder and a fully extended elbow.
3. **Disability of the Arm, Shoulder, and Hand (DASH) scores**: This is a 30-item self-reporting scoring system with each item in 5 categories. The score ranges from 0 for no disability to 100 for severe disability. It is commonly used to assess the function of the upper limb objectively.

### Statistical analysis

The statistical analysis was carried with SSPS (Statistical Package for the Social Sciences) version 27. Frequencies and percentages were used to describe the study population. As for the continuous data set, the means, standard deviation, and range were reported. Chi-Square statistics were used to describe the association between piano key sign and union by fracture site. The association of DRUJ ballottement, Piano key sign and radiographical assessment by fracture site were also determined using Chi-square. Fisher-Exact statistics were used as an alternative for Chi-Square statistics when cells have less than five counts. An independent t-test was performed to compare the differences in union status between DASH, VAS, VASW, grip strength and to compare the differences in union status between the range of movements (ROM). The statistical significance is known as P-value less than 0.05 (p<0.05).

## Results

A total of 74 patients treated distal end of radius fractures were interviewed, out of which 56 (76%) patients had concomitant ulna styloid fractures. The mean age was 32 years (range: 18–69; SD: ± 12.7). There were 43 (76.8%) men and 13 (23%) women in this study. The right wrist was involved in 32%, the left wrist in 68% of subjects and 91% were right-hand dominant. The mean time from injury was 18.7 months (range: 6–84; SD: ± 13.3).

The most common type of fracture was Frykman 2 (35.7%), followed by type 6 (25%), type 8 (23.2%) and type 4 (16.1%). All the patients had closed fractures; 60.7% were base, and 39.3% were tip fractures. Half (50%) of the distal radius fractures were treated with casting, 48.3% were treated with plating, and 1.8% with external fixation. The mean period of casting for patients treated conservatively was 7.67 weeks (range: 4–16; SD ± 3.1). The ulna styloid was united in 35.7% of patients, and non-united in 64.3%; 50% of the tip fractures had united, and only 26.5% of the base fractures had united.

The average range of movement (ROM) compared to the opposite wrist is given in Table 1. The mean wrist extension is 81.6%±20.1; wrist flexion is 77.8%±22.7; Ulna deviation is 71.0%±22.2; Radial deviation is 75.4%±21.4; Pronation is 84.0%±20.5, and Supination is 88.6%±18.0. The mean percentage of movement for subjects with ulna styloid union is as follows; wrist extension is 82.4%±18.2; wrist flexion is 76.7%±25.9; Ulna deviation is 71.3%±23.2; Radial deviation is 73.5%±18.3; Pronation is 86.1%±17.8 and Supination is 86.9%±18.2 and the mean percentage of movement for patients with ulna styloid nonunion is; wrist extension 81.1%±18.2; wrist flexion 78.3%±21.1; Ulna deviation is 70.9%±21.9; Radial deviation is 76.4%±23.1; Pronation is 82.8%±22.0 and Supination is 89.5%±18.0. The difference is statistically insignificant.

**Table 1:** Shows the mean wrist movement for a different mode of treatment.

Treatment	Extension (%)	Flexion (%)	Ulna Deviation (%)	Radial Deviation (%)	Pronation (%)	Supination (%)
Total	81.6±20.1	77.8±22.7	71.0%±22.2	75.4%±21.4	84.0%±20.5	88.6%±18.0
Ulna Styloid united	82.4±23.7	76.7±25.9	71.3±23.2	73.5±18.3	86.1±17.8	86.9±18.2
Non-union Ulna styloid	81.1±18.2	78.3±21.1	70.9±21.9	76.4±23.1	82.8±22.0	89.5±18.0
P value	0.83	0.80	0.95	0.63	0.57	0.60

The Ballottement test was positive in 23.2% of subjects. This consists of 20% of those with united ulna styloid and 25% with ulna styloid non-union (p = 0.470). 32% of subjects with non-union base (p = 0.225) and 9% of non-union of the tip of the ulna (p = 0.293) had ballottement positive (Table 2). All are statistically insignificant.

**Table 2:** Shows DRUJ ballottement with radiographical assessment and site of the fracture.

Site			Radiographical assessment		Total
			NO	YES	
Tip	DRUJ ballottement	NO	10(90.9%)	8(72.7%)	18(81.8%)
		YES	1(9.0%)	3(27.3%)	4(18.2%)
	Total		11	11	22
Base	DRUJ ballottement	NO	17(68%)	8(88.9%)	25(73.5%)
		YES	8(32%)	1(11.1%)	9(26.5%)
	Total		25	9	34
Total	DRUJ ballottement	NO	27(75%)	16(80%)	43(76.8%)
		YES	9(25%)	4(20%)	13(23.2%)
	Total		36	20	56

The Piano key sign was positive in 16.7% of subjects with ulna styloid non-union and 15% with united ulna styloid (p = 0.595). 24% of non-union of base fractures (p = 0.386) and none of the non-union of the tip fractures were associated with a positive piano key sign (Table 3). All are statistically insignificant.

**Table 3:** Shows the relationship of the piano key sign to the site of fracture and union.

Site			Ulna styloid Union		Total
			NO	YES	
Tip	Piano key sign	NO	11(100%)	9(81.8%)	20(90.1%)
		YES	0	2(18.2%)	2(9.9%)
	Total		11	11	22
Base	Piano key sign	NO	19(76%)	8(88.9)	27(79.4%)
		YES	6(24%)	1(11.1%)	7(20.6%)
	Total		25	9	34
Total	Piano key sign	NO	30(83.3%)	17(85%)	47(83.9%)
		YES	6(16.7%)	3(15%)	9(16.1%)
	Total		36	20	56

All the displacements were dorsal except in 1 case of united ulna styloid base fracture is volar. 11.1% of the subjects with ulna styloid non-union had DRUJ displacement compared to 25% with the united ulna styloid. The mean displacement of the non-union fractured ulna styloid is 1.88mm (SD±1.08, Range: 0.20–4.60mm).

As for the ulnar sided wrist pain, the mean VAS score at rest is one and at work is 5. The mean VAS score for ulna styloid union at rest is 0, and non-union is 1.0 (p = 0.16). The mean VAS at work is 5 for both with ulna styloid union and non-union (p = 0.6). Both are statistically insignificant.

The mean grip strength for all the subjects at the affected limb is 18.0 kg (SD±9.5), and the opposite is 32.7 kg (SD±11.2). The subjects had 56% of their regular grip. The grip was 52.9% in the cases of ulna styloid union and 57.7% in the cases of ulna styloid non-union (p = 0.52). This was statistically insignificant.

The mean DASH score for the study group is 44.68 (SD±20.5). The functional score for the united ulna styloid is 50.0 (SD±22.3), and for the non-union ulna styloid is 41.7 (SD±19.0). This is statistically insignificant (p = 0.145).

## Discussion

Hauck RM et al. reported the incidence rate of the distal end of radius fracture associated with a styloid ulna fracture at nearly 76 cases per 100 distal radial fractures. Ulna styloid fracture rarely occurs in isolation [14,15]. Frykman’s type 2 fractures had the highest incidence in our study among the types of distal end radius fractures with ulnar styloid involvement, consistent with the reported incidence in literature [16]. Ulnar styloid fractures were subdivided into tip and base fractures by Zenke Y et al. [17]. In our study, there was a higher incidence of the base fractures, where half of the tip and quarter of the base fractures had united.

Almost all ulna styloid fractures are associated with distal radius fractures or triangular fibrocartilage complex (TFCC) injuries. Thus, an algorithm to treat ulna styloid fracture is difficult to construct without considering the associated injuries. Nevertheless, the decision to treat or not to treat ulna styloid fractures surgically should be made as it should be performed simultaneously during the radius fixation. The fixation of ulna styloid is technically demanding and potentially causes unwanted complications; hardware-related problems requiring another surgery for removal [17], and dorsal sensory branch of ulna nerve injuries [18]. Therefore, non-surgical treatment for ulna styloid fractures is not a wrong choice.

In this study, we found 76% of distal radius fractures to have concomitant ulna styloid fractures. We did not identify any patient with isolated ulna styloid fracture, probably because patients did not seek initial treatment as they had mild symptoms or missed diagnosis during the first presentation. All treatments were focused on radius fractures, and ulnar styloid was neglected. Non-union ulna styloid was seen in 64.3% of the total participants. The range of movement (ROM) of the affected wrist is almost similar in both ulna styloid union and non-union groups. Therefore, the union status of the ulna styloid does not influence eventual wrist range of movement.

The parameters for DRUJ instability investigated in this study are the clinical tests such as the Ballottement test, Piano key sign, and the radiological ulna displacement. We found that the Ballottement test was positive in 23.2% of subjects. Both the united and non-united ulna styloid groups had a similar incidence of positive Ballottement test. A more significant percentage (three-fold) of subjects with the non-union base than non-union of the tip of ulna had ballottement positive. The Piano key sign was positive in an almost equal percentage of subjects with union and non-union of the ulna. They were mainly related to the base of ulna fractures. This is in keeping with what is reported by May M M et al. [2], who proposed that the base of ulna styloid fracture has been shown to contribute to DRUJ instability in distal radius fractures. The patients with the base of ulna styloid fracture constitute a more significant portion of samples in our study with a positive result in DRUJ stability assessment, namely the Piano Key sign and DRUJ ballottement test. However, no statistically significant difference was found between wrist stability and the type of ulna styloid fracture; this is in keeping with the finding from Kim K.W et al [19] who found that DRUJ instability was independent of the ulna styloid fracture location. Furthermore, Clementsen [20] reported that distal radius fractures associated with ulna styloid fracture do not affect the patient-related outcome, range of motion, or grip strength; therefore, they suggest that the ulna styloid fracture can be left untreated in cases of distal radius fracture.

More subjects with united ulna styloid had DRUJ displacement radiologically compared to the non-union in our study subjects. This is probably contributed by the malunited distal radius fractures rather than the ulna styloid in isolation. All the displacements were dorsal except in 1 case of united ulna styloid base fracture.

As for functional assessment, ulna-sided pain was assessed vis the Visual Analog score both at rest and at work, and The mean VAS score at rest is one and at work is 5. The mean VAS score for ulna styloid union at rest is 0, and non-union is 1.0 (p = 0.16). The mean VAS at work is 5 for both ulna styloid union and non-union (p = 0.6). Both are statistically insignificant. Therefore, the status of the union of the ulna styloid did not influence the severity of ulna-sided pain both at rest and at work.

The mean grip strength for our subjects is slightly above half compared to the opposite hand. We found that grip strength between 2 groups of patients with or without ulna styloid union is very similar and the difference was insignificant. Similarly, we found no statistical difference in the DASH score between united and non-united ulna styloid fractures.

The union status of an ulna styloid process fracture has no impact on the wrist outcome in terms of functional and clinical assessment of wrist stability based on our study result. Several studies have shown a similar outcome [17,21,22]. Regarding the findings above, many researchers are looking into the contribution of the soft tissue component of the wrist to the outcome of ulnar-sided pain. They routinely employ arthroscopy as part of the initial assessment in fracture fixation [17].

Previous studies had emphasized the correlation between ulna styloid fractures and DRUJ instabilities [2,14]. They found a higher risk of DRUJ instability in ulna styloid base fractures and fracture displacement of > 2mm. However, the surgical treatment of radius fractures and ulna styloid fractures was not standardized. Furthermore, the non-union rate of the ulna styloid was not mentioned.

Zenke et al. [17] and Souer et al. [21] managed to standardize their participants; all distal radius fractures were treated with volar locking plates. Both studies claimed that the clinical and functional outcome was good despite not fixing the ulna styloid base fractures. However, the authors failed to include DRUJ instability in their assessment. This view is echoed by Dennison DG [23]. The results from our study are primarily in line with the above hypothesis.

Chen, A.CY et al [24] compared early and late fixations of ulna styloid fractures using the QuickDASH scoring, the Grip strength, the range of movement and Pain via VAS scoring system. They found that patients who had early ulna fixation had better function compared to those who underwent late fixations. If we compare their overall results of surgical fixation of the ulna styloid, their patient cohort had a better overall grip strength (33.2 ± 7.3 kg vs. 18.0 kg ± 9.5). The wrist flexion (75.9 ± 5.8. vs 77.8%±22.7) and wrist extension (80.3 ± 6.8 vs. 81.6%±20.1) are similar to our study but the Pronation (75 ± 8.7 vs. 84.0%±20.5) and Supination (70 ± 13.2 vs. 88.6%±18.0) yield poorer results. We are unable to compare the functional score between both studies as the scoring system used differs (QuickDASH vs. DASH). Furthermore, their study is based on patient with isolated ulna styloid fractures with established DRUJ instability, where as our patients consist of distal radius fractures with concomitant ulna styloid fractures. That’s would probably explain the better grip strength.

These contradicting findings and suggestions proposed regarding ulna styloid fracture treatment by many works of literature are due to heterogeneity of study design, mechanism of injury, associated injuries, treatment of distal radius fractures, and treatment of ulna styloid fractures. To date, there is no optimal management plan for ulna styloid fracture.

In our study, all the distal radius fractures were united, and patients achieved a good range of motion at final follow-up. However, most had ulna-sided wrist pain during work, DRUJ instability, reduced grip strength, and functional disabilities. There was no significant difference in clinical, functional, and radiographic findings between united and non-union ulna styloid. The possible reason contributing to the issues above is probably the TFCC injuries which were not addressed. TFCC injuries are highly likely as the deep limb of TFCC attaches at the fovea, whereas the superficial limb of TFCC attaches at the ulna styloid [25]. Avulsion of the base of the ulna styloid process may indicate avulsion or tear of TFCC [2,26]. However, the footprint of TFCC insertion at the fovea is broad that it may not cause total avulsion of TFCC [25]. The majority of the patients’ wrists were immobilized in splint or slab, which probably allowed healing of TFCC tears in these patients.

The limitations of this study are the small sample size and retrospective study design. Furthermore, the distal radius fractures were treated differently. MRI and Ct scans are a more accurate method of radiological assessment of DRUJ. This was not carried out in this study due to cost issues and this is a retrospective study.

In conclusion, we believe that all ulna styloid fractures found concomitant with distal radius fractures do not seem to contribute to the DRUJ stability as we found that the status of the union of the ulna styloid and the site of the ulna styloid fracture (tip or base) did not have a bearing on the range of movement and functional status of the affected wrist. Patients with distal radius fractures should be assessed for DRUJ instability and the positive cases managed with a temporary DRUJ immobilization, which might allow for TFCC recovery. Further management should be considered for cases with chronic instability with an MRI of the wrist and arthroscopy.
